# Digital technologies for sustainable food supply chains: a scoping review of impacts on food safety, loss reduction, and implications for nutritional security

**DOI:** 10.3389/fnut.2026.1796752

**Published:** 2026-03-31

**Authors:** Shuang Li, Haoyang Li, Tianyi Kong

**Affiliations:** 1 Department of Economic Management, Beijing Jiaotong University, Beijing, China; 2 School of Accounting, Guangzhou College of Technology and Business, Guangzhou, China; 3 Division of the Humanities, University of Chicago, Chicago, IL, United States

**Keywords:** digital technology, food supply chain, blockchain, Internet of Things, artificial intelligence, food safety, food loss, nutritional security

## Abstract

Digital technologies such as blockchain, Internet of Things (IoT), and artificial intelligence (AI) are increasingly applied in food supply chains (FSCs) to enhance traceability, monitoring, and quality detection. However, whether these technological advancements ultimately contribute to improved nutritional security remains unclear. This scoping review systematically examined the application of digital technologies across food supply chains and assessed their impacts on food safety, food loss reduction, and nutritional outcomes. Following PRISMA guidelines, we searched Web of Science, Scopus, and PubMed for empirical studies published between 2015 and 2025, with supplementary searches conducted in the Cochrane Library and Global Health databases to ensure comprehensive coverage of nutrition-related evidence. Ultimately, 46 studies were included for analysis. Results revealed that blockchain (41.3%), IoT (26.1%), and AI/machine learning (19.6%) constitute the primary technological approaches, with storage and transportation being the most concentrated application areas (41.3%). Evidence for positive impacts on food safety and loss reduction is substantial, with 89% and 83% of relevant studies reporting favorable outcomes, respectively. However, only 6.5% of included studies directly measured nutrition-related outcomes, representing a critical evidence gap. This asymmetric evidence distribution suggests that while digital technologies demonstrate clear value in improving supply chain efficiency and food safety, the pathway from technological performance to nutritional improvement remains largely unverified. Future research should incorporate nutrition indicators into evaluation frameworks and prioritize studies in low-income countries where food loss and nutritional insecurity are most severe.

## 1 Introduction

In 2016, retail giant Walmart completed an experiment to trace the origin of a package of mangoes. Using traditional methods, this process took nearly 7 days; with blockchain technology, the same trace required only 2.2 s. This case has been widely cited as a landmark event in the digital transformation of food supply chains (FSCs) (1). However, when we ask a more fundamental question, the answer becomes far less clear: can these technologies actually help people eat more safely and more nutritiously? Approximately one-third of the world's food is lost or wasted each year on its journey from farm to table (2), while more than 800 million people still face hunger and billions suffer from micronutrient deficiencies, often termed “hidden hunger” (3). Blockchain can trace a package of mangoes to its source, Internet of Things (IoT) sensors can monitor cold chain temperatures, and artificial intelligence (AI) can predict shelf life, but can these technologies truly reduce nutrient loss and improve dietary accessibility? This is the core question that this study attempts to answer.

Global food supply chains (FSCs) are facing unprecedented sustainability challenges. As globalization accelerates and market complexity increases, FSCs have evolved into complex networks involving multiple stages and participants, with every link from farm to table facing tests of efficiency, safety, and sustainability (4). Frequent food safety incidents represent a primary challenge confronting current supply chains, as contamination, adulteration, and recall events not only threaten consumer health but also severely damage consumer trust in the food system (5). In the European Union, cereals, fruits, and vegetables are the food categories responsible for the highest amounts of food waste, with the consumption stage being the most wasteful for most food categories (2). Losses during transportation are equally significant; research indicates that even in developed countries, a considerable proportion of dairy products fail to reach consumers due to various reasons (6). These losses and waste have profound indirect impacts on nutritional security: when food is lost in the supply chain, it represents not only a loss of calories but also a loss of valuable nutrients, with particularly significant effects on vulnerable populations in low- and middle-income countries (7). In fact, reducing postharvest losses can significantly improve access to nutritious food for populations in these regions, especially for nutritionally vulnerable groups such as children under five and pregnant women (3, 8). In the context of global food system transformation, although dietary affordability has improved, food systems of all types are still falling short of delivering optimal outcomes in terms of nutrition and health, environmental sustainability, and inclusion and equity (9).

The rapid development of digital technologies offers new possibilities for addressing these challenges. In recent years, the application of blockchain, IoT, AI, and machine learning (ML) in FSCs has experienced explosive growth (10, 11). Blockchain technology, with its characteristics of immutability and transparency, is widely considered capable of providing a reliable technical foundation for food traceability, enabling tracking from source to final consumer (12). IoT technology, through the deployment of sensor networks across various supply chain stages, enables real-time monitoring of critical parameters such as temperature, humidity, and gas composition, which is particularly important for cold chain management of perishable foods (13, 14). AI and ML technologies have demonstrated significant potential in food quality detection, freshness classification, and demand forecasting, enabling rapid, non-destructive assessment of food quality (15, 16). The integrated application of these technologies is driving the transformation of FSCs toward intelligence and digitalization, forming a new paradigm of “Food Traceability 4.0” (11). In precision agriculture, the combination of IoT devices and remote sensing technology enables farmers to obtain real-time data on crop health, soil conditions, and environmental factors, thereby optimizing decisions regarding irrigation, fertilization, and pest control (17, 18). In the evolution from Industry 4.0 to Industry 5.0, these technologies are being further integrated with emerging technologies such as generative AI, digital twins, and collaborative robots, promising to bring deeper transformations to the food industry (19).

However, existing research has notable gaps and limitations. First, most studies on the application of digital technologies in FSCs focus on technical feasibility and system framework design, while evaluation of actual deployment effects remains relatively insufficient (20). Second, although blockchain and similar technologies are widely believed to enhance supply chain transparency and efficiency, their large-scale application in FSCs still faces numerous boundary conditions, including challenges related to standardization, governance mechanisms, and organizational coordination (5). More critically, existing research on the connection between digital technologies and nutritional security is almost non-existent. This absence is striking given that the nutrition-sensitive value chain framework explicitly calls for tracing food system interventions beyond production efficiency to their impacts on dietary quality and nutritional outcomes (21, 22). Value chain approaches have long been used to enhance producer livelihoods, yet they have rarely been employed as tools to achieve nutritional goals (21). If digital technologies genuinely reduce postharvest losses and enhance food safety, such improvements should theoretically contribute to better nutrient retention and dietary accessibility, but this assumed pathway has never been empirically tested. Furthermore, the application of digital technologies faces the challenge of the digital divide: research shows that the proportion of small-scale farms with access to 3G or 4G network services is far lower than that of large-scale farms, and regions with severe yield gaps, high climate stress, and food insecurity are precisely those with the poorest network coverage (23). This means that the benefits brought by digital technologies may not equitably reach those who need them most.

**Figure 1:**
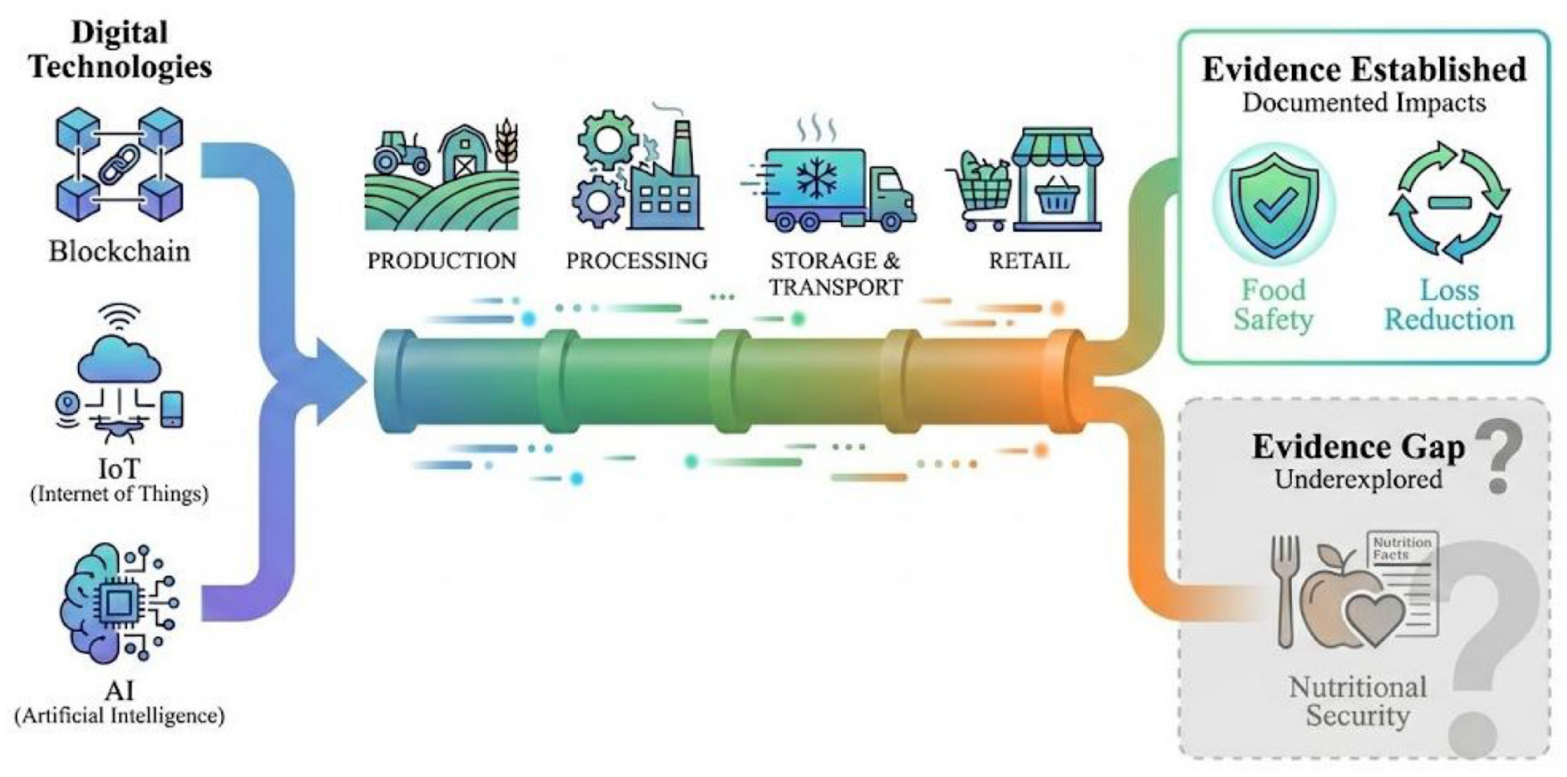
Conceptual framework of digital technology-driven sustainable food supply chains.

Based on this background, this study aims to systematically review the current status of digital technology applications in FSCs and their impacts on food safety and loss reduction through a scoping review methodology, while also assessing the implications of existing evidence for nutritional security. As shown in Figure 1, the conceptual framework of this study depicts the pathways through which digital technologies transmit effects across different levels through FSCs, where food safety and loss reduction already have relatively substantial evidence support, while evidence at the nutritional security level remains to be explored. Specifically, this study attempts to answer the following four questions: (1) What applications do digital technologies such as blockchain, IoT, and AI have across different stages of FSCs? (2) What impacts do these technologies have on food safety and quality assurance? (3) What contributions do these technologies make to reducing food loss and waste? (4) How many studies in the existing evidence directly address nutritional outcomes? By answering these questions, this study aims to provide policymakers, supply chain managers, and researchers with comprehensive evidence on the effects of digital technology applications, while identifying critical evidence gaps in the transmission pathway from technical efficiency to nutritional security and pointing directions for future research.

## 2 Methods

### 2.1 Study design and search strategy

This study employed a scoping review methodology to systematically examine the applications of digital technologies in FSCs and their impacts on food safety, loss reduction, and nutritional security. Scoping reviews are particularly suitable for emerging research fields, as they can effectively map the current state of research, identify key concepts, and reveal evidence gaps (24). Unlike systematic reviews that focus on evaluating the effectiveness of specific interventions, scoping reviews are more appropriate for answering questions such as “what has been studied in a given field” and “how is the evidence distributed,” which aligns well with the objectives of this study. This study followed the Preferred Reporting Items for Systematic Reviews and Meta-Analyses (PRISMA) extension for scoping reviews (25).

Literature searches were conducted in three academic databases: Web of Science, Scopus, and PubMed. The selection of these three databases was based on the following considerations: Web of Science and Scopus are the most comprehensive multidisciplinary indexing databases, capable of retrieving research across interdisciplinary fields including food science, supply chain management, and information technology; PubMed, as an authoritative database in the biomedical field, helps identify research related to nutrition and food safety, ensuring that the nutritional perspective of this review is adequately represented. The search time frame was set from January 2015 to December 2025, covering the main period from the emergence of research on digital technology applications in FSCs to the present. The search strategy was constructed using a three-layer keyword combination corresponding to the technology layer, supply chain layer, and outcome layer, connected by the Boolean operator “AND” between layers and “OR” within each layer. Specific search terms are presented in Table 1. The search scope was limited to title, abstract, and keyword fields. Additionally, snowball searching of reference lists from included studies was conducted to supplement potentially missed relevant literature. Given that this review explicitly addresses the nutritional security dimension, a supplementary search was conducted in the Cochrane Library and Global Health databases to ensure comprehensive coverage of nutrition-related evidence. This supplementary search employed the same three-layer keyword structure, with particular emphasis on nutrition-specific terminology including “bioavailability,” “micronutrient retention,” “vitamin loss,” and “dietary diversity” combined with digital technology and supply chain terms. This additional search aimed to verify whether empirical studies examining the intersection of digital technologies and nutritional outcomes existed outside the scope of the primary databases.

**Table 1:** Search strategy keywords.

Layer	Search terms
Technology layer	“digital technolog^*^” OR “blockchain” OR “Internet of Things” OR “IoT” OR “artificial intelligence” OR “machine learning” OR “deep learning” OR “smart agriculture” OR “precision agriculture” OR “RFID” OR “sensor network^*^” OR “big data” OR “computer vision”
Supply chain layer	“food supply chain” OR “agri-food” OR “agrifood” OR “cold chain” OR “traceability” OR “food logistics” OR “food processing” OR “food storage” OR “food distribution” OR “postharvest” OR “post-harvest” OR “farm to fork”
Outcome layer	“food safety” OR “food security” OR “food loss” OR “food waste” OR “food quality” OR “freshness” OR “shelf life” OR “spoilage” OR “nutritional” OR “nutrient retention” OR “nutrient loss” OR “nutrient degradation” OR “micronutrient” OR “bioavailability” OR “vitamin retention” OR “nutritional quality” OR “dietary quality” OR “contamination”

### 2.2 Inclusion and exclusion criteria

To ensure the relevance and quality of included studies, this study established clear inclusion and exclusion criteria, detailed in Table 2. Inclusion criteria were: (1) the study involved the application of digital technologies such as blockchain, IoT, and AI in FSCs; (2) study types included case studies, technology validation, prototype testing, field experiments, or survey research; (3) the study reported results or indicators related to food safety, quality, loss, waste, or nutrition (nutrition-related outcomes were defined as any measurement of nutrient content, retention, or degradation such as vitamin loss, micronutrient bioavailability, or nutritional quality changes, as well as downstream indicators including dietary diversity, dietary accessibility, or population nutritional status); (4) published in English in peer-reviewed journals with full text available. Exclusion criteria were: (1) pure technology development studies that only described algorithms or system architectures without supply chain application scenarios; (2) reviews, editorials, commentaries, conference abstracts, or book chapters; (3) studies that only discussed technical feasibility without reporting any outcome data; (4) non-English literature. Regarding technology validation studies, although such studies may only have been tested in laboratory environments or on standard datasets, they can reflect technology maturity and application potential. They are valuable for comprehensively mapping the development status of this field and were therefore included in this review.

**Table 2:** Inclusion and exclusion criteria.

Criteria type	Inclusion criteria	Exclusion criteria
Research topic	Involving digital technology applications in FSCs	Pure technology development without supply chain application scenarios
Study type	Case studies, technology validation, prototype testing, field experiments, survey research	Reviews, editorials, commentaries, conference abstracts, book chapters
Outcome reporting	Reporting results related to food safety, quality, loss, waste, or nutrition	Only discussing technical feasibility without outcome data
Language and publication format	English peer-reviewed journal articles with full text available	Non-English literature, gray literature, full text unavailable

### 2.3 Literature screening and data extraction

Literature screening was conducted following a two-stage process. The first stage involved title and abstract screening, excluding clearly irrelevant literature based on the inclusion and exclusion criteria. The second stage involved full-text screening, where literature that passed the initial screening underwent full-text reading to further assess whether it met the inclusion criteria. During the screening process, questionable literature was decided upon through discussion and consultation, and all excluded literature had exclusion reasons recorded to ensure transparency and reproducibility of the screening process. Literature management was performed using EndNote software for deduplication and organization, and the screening process and the number of literature at each stage are presented as a PRISMA flow diagram in the results section.

Data extraction was performed using a pre-designed standardized form, with extraction content covering five aspects: (1) basic information, including authors, publication year, country or region where the study was conducted, and publication journal; (2) study characteristics, including study design type, sample size or study setting, and food types involved; (3) technology information, including the types of digital technologies applied and specific technical solutions; (4) supply chain information, including the supply chain stages involved in technology application and descriptions of application scenarios; (5) outcome information, including main outcome indicators, quantitative data, and effect direction.

### 2.4 Data synthesis methods

This study employed a combination of descriptive statistics and thematic analysis for data synthesis. Due to the substantial heterogeneity in study design types, technical solutions, application scenarios, and outcome indicators across this field, quantitative meta-analysis was not appropriate. Descriptive statistics were used to present the distribution of basic characteristics of included studies, including publication year trends, geographic distribution, composition of study design types, distribution of technology types, and coverage of supply chain stages. Thematic analysis was organized according to the four research questions of this study: first synthesizing and analyzing the application of digital technologies across various FSC stages, then reviewing the evidence of technology impacts on food safety and food loss reduction respectively, and finally assessing the status of evidence regarding nutrition-related outcomes in existing research. For effect direction, counting methods were used to tally the number of studies reporting positive effects, no significant differences, and unclear reporting, to intuitively present the distribution of existing evidence. This study did not conduct formal methodological quality assessment of included studies, which is consistent with the positioning of scoping reviews, as the main purpose of scoping reviews is to map the scope of evidence and identify research gaps rather than to systematically evaluate the strength of evidence (26).

## 3 Results

### 3.1 Literature screening and overview of included studies

**Figure 2:**
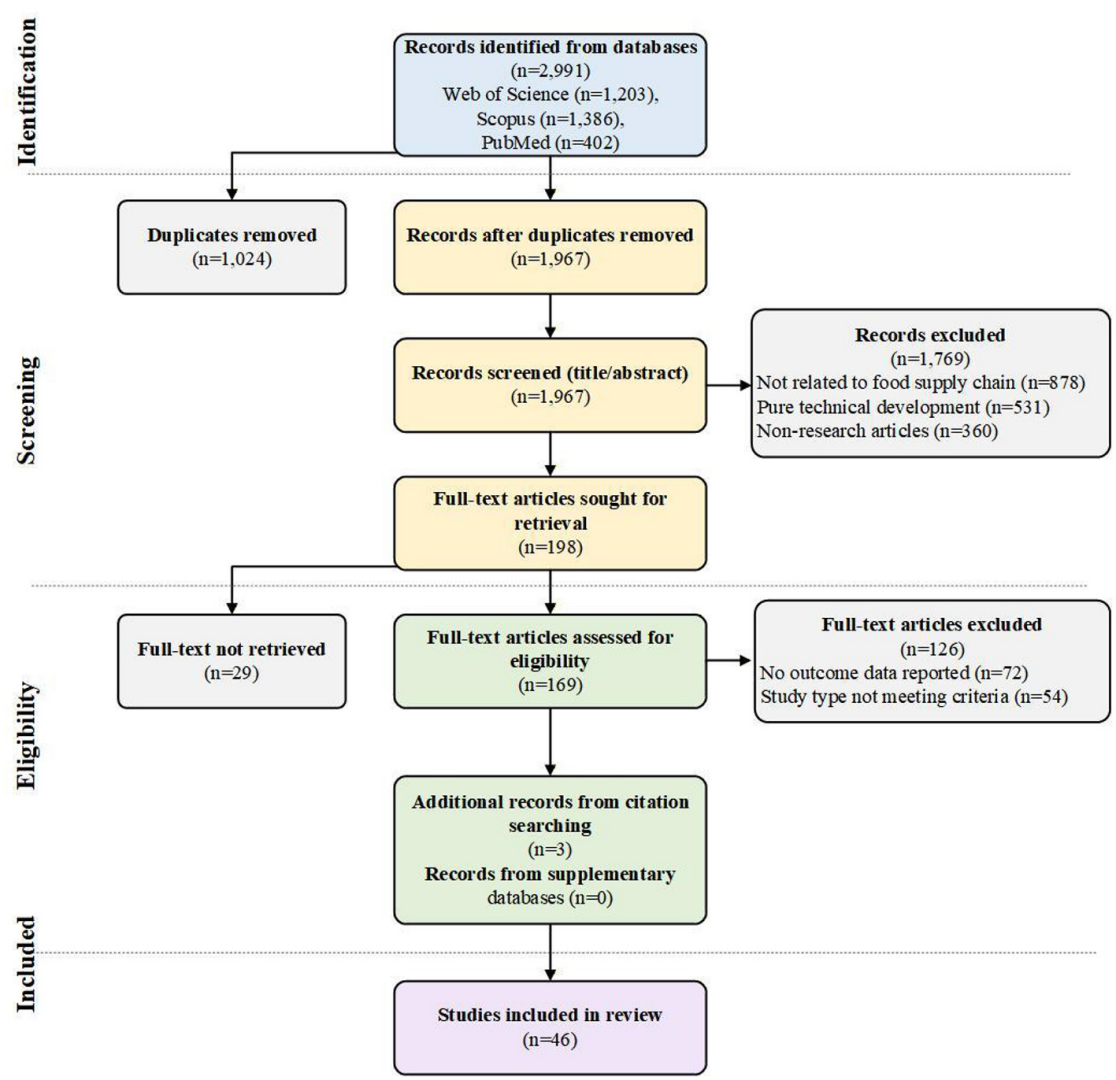
PRISMA flow diagram of literature screening.

Database searches retrieved a total of 2,991 articles, including 1,203 from Web of Science, 1,386 from Scopus, and 402 from PubMed. After removing 1,024 duplicate articles using EndNote software, 1,967 articles remained. Following the first stage of title and abstract screening, 1,769 articles were excluded, with main reasons for exclusion including: not related to FSCs (*n* = 878), pure technology development studies (*n* = 531), and non-research articles (*n* = 360). In the second stage, full-text retrieval was attempted for 198 articles, of which 29 could not be retrieved. Full-text screening of the remaining 169 articles resulted in the further exclusion of 126 articles, with main reasons for exclusion being: only discussing technical feasibility without reporting outcome data (*n* = 72) and study type not meeting inclusion criteria (*n* = 54). An additional three articles meeting the criteria were included through snowball searching. The supplementary search in Cochrane Library and Global Health databases yielded 127 and 284 records respectively; however, after applying the same screening criteria, no additional studies meeting the inclusion criteria were identified. Ultimately, 46 articles were included for comprehensive analysis, with the specific screening process shown in Figure 2.

The basic characteristics of included studies are presented in Table 3. Regarding publication time, included articles showed a clear growth trend, with studies published between 2015 and 2019 accounting for 23.9%, while those published between 2020 and 2025 accounted for 76.1%, reflecting the rapid rise in research activity in this field. Regarding geographic distribution, the Asian region contributed the most studies (41.3%), with China, India, and Vietnam being the main source countries; the European region accounted for 37.0%, primarily from Italy, the Netherlands, and the United Kingdom; North America and Oceania accounted for 13.0 and 8.7%, respectively. Regarding study design types, real-world supply chain deployment cases accounted for 50.0%, technology validation studies for 39.1%, and survey research for 10.9%. Regarding digital technology types involved, blockchain-related studies were most numerous (41.3%), followed by IoT (26.1%), AI and ML (19.6%), and multi-technology integrated systems (13.0%). Regarding supply chain stages, storage and transportation was the most concentrated area of research (41.3%), followed by multi-stage comprehensive studies (28.3%), production (15.2%), processing (8.7%), and retail (6.5%). Regarding food types, studies covered multiple categories including fruits and vegetables, meat, dairy products, aquatic products, and grains, with fruits and vegetables being the most studied, reflecting the urgent need for digital management of perishable foods.

**Table 3:** Basic characteristics of included studies (*n* = 46).

Characteristic	Category	Number	Percentage
Publication year	2015–2019	11	23.9
	2020–2022	20	43.5
	2023–2025	15	32.6
Region	Asia	19	41.3
	Europe	17	37.0
	North America	6	13.0
	Oceania	4	8.7
Study design	Real-world deployment cases	23	50.0
	Technology validation	18	39.1
	Survey research	5	10.9
Technology type	Blockchain	19	41.3
	IoT	12	26.1
	AI/ML	9	19.6
	Integrated systems	6	13.0
Supply chain stage	Production	7	15.2
	Processing	4	8.7
	Storage and transportation	19	41.3
	Retail	3	6.5
	Multiple stages	13	28.3

### 3.2 Mapping of digital technology applications

Included studies covered major digital technology types including blockchain, IoT, AI, and ML, with these technologies exhibiting differentiated application characteristics across different stages of FSCs.

Blockchain technology applications were primarily concentrated in traceability and transparency enhancement, recording information throughout the entire process from production to consumption through distributed ledgers. A case study of the pork supply chain in Vietnam demonstrated how blockchain can achieve end-to-end traceability from farms to retail terminals (27), while a traditional bakery in Italy combined blockchain with IoT sensors to achieve complete records of raw material sources, production environments, and transportation conditions (28). In the dairy sector, blockchain has been applied to ensure product origin certification and quality traceability (29). Research on the Thai broiler supply chain indicated that compared to traditional centralized systems, blockchain infrastructure can reduce total costs by approximately 43% (30).

IoT technology applications in FSCs were primarily manifested in real-time monitoring and data collection, playing a critical role particularly in cold chain management. Research has shown that simultaneous deployment of Radio Frequency Identification (RFID) tags and Wireless Sensor Networks (WSN) in commercial cold storage facilities can achieve three-dimensional spatial monitoring of storage environment temperature and humidity, and can estimate energy consumption and product moisture loss based on this data (31). A frozen food company in the United Kingdom achieved real-time monitoring of storage conditions through IoT sensor deployment, effectively identifying optimal and non-optimal storage conditions (32). In agricultural product transportation monitoring, IoT devices can continuously track cargo location and environmental parameters, automatically triggering alerts when anomalies are detected (33). Recent research has further extended IoT services to monitoring the entire agri-food supply chain ecosystem, covering the full process from packaging to last-mile delivery (34).

AI and ML technologies were primarily applied in food quality detection, freshness assessment, and demand forecasting. In quality detection, image classification models based on Convolutional Neural Networks (CNN) have been widely used for automatic identification of fruit freshness, with reported classification accuracies generally exceeding 95% (35). For aquatic products, machine vision-based freshness assessment systems can achieve non-destructive detection by analyzing fish body image features (36). In cold chain logistics scenarios, ML algorithms have been used to integrate multi-source sensor data to predict blueberry freshness, with research showing that Support Vector Machine (SVM) models can achieve prediction accuracy of 94.01%, outperforming traditional Arrhenius equation methods (37). Additionally, ML has been applied to agricultural yield prediction and planting decision optimization (38).

Some studies explored the integrated application of multiple technologies. The combination of blockchain and IoT was the most common integration pattern, with IoT devices responsible for automatically collecting data across various supply chain stages while blockchain ensures the immutability and traceability of this data (39, 40). More comprehensive technical frameworks have integrated blockchain, ML, cloud computing, and QR code technology for food waste management in smart city contexts (41). A distinct integration approach was demonstrated by Defraeye et al. (42), who developed a physics-based digital twin model that combines real-time sensor data with mechanistic simulations of heat transfer and biochemical quality evolution, enabling prediction of both physical and nutritional quality changes in mango fruit during refrigerated transport. Table 4 summarizes the main application scenarios of different technology types across various supply chain stages.

**Table 4:** Mapping of digital technology applications across FSC stages.

Technology type	Production stage	Processing stage	Storage and transportation stage	Retail stage
Blockchain	Farm information recording, certification management	Processing traceability	Logistics tracking, cold chain records	Consumer traceability queries, anti-counterfeiting verification
IoT	Environmental monitoring, precision irrigation	Processing parameter monitoring	Temperature and humidity monitoring, location tracking	Smart shelves, inventory monitoring
AI/ML	Yield prediction, pest and disease identification	Quality grading, defect detection	Freshness prediction, route optimization	Demand forecasting, dynamic pricing

### 3.3 Impacts on food safety and quality

Twenty-seven of the included studies reported impacts of digital technologies on food safety and quality. Overall, evidence presented a consistent positive trend, although evidence maturity varied across technology types: blockchain applications in traceability were the most widespread with consistent effects, AI demonstrated high accuracy in detection but mostly through laboratory validation, and IoT monitoring applications had accumulated more cases in real-world settings.

Regarding traceability capabilities, the application effects of blockchain technology were consistently supported by studies across different regions and product categories. A pilot study in the Netherlands was among the first to confirm that blockchain can provide shared, immutable transaction records for all supply chain participants (43), a core finding that has been repeatedly observed in subsequent research: multi-case analysis of organic food supply chains showed that blockchain helps verify the authenticity of certification information (44), applications in the Italian Fontina cheese and dairy product supply chains achieved end-to-end digital traceability from pasture to consumer (29, 45), and the Vietnamese pork supply chain and Italian traditional bread production similarly validated the technical feasibility of full-chain traceability (27, 28). A case study of the U.S. egg supply chain demonstrated the commercial application of blockchain combined with IoT to achieve “farm to fork” tracking (1). Notably, Stranieri et al. (46), through systematic interviews with three supply chains led by European retailers, was among the few studies to assess organizational-level effects, finding that blockchain implementation indeed improved information accessibility and sharing, and enhanced management of behavioral uncertainty. Regarding cost-effectiveness, Pongnumkul et al. (30), based on Monte Carlo simulation analysis of the Thai broiler supply chain, showed that blockchain infrastructure total costs can be reduced by approximately 43% compared to traditional centralized systems. Additionally, smart contract-based traceability frameworks have been validated in Chinese agricultural enterprises (47) and rice supply chains (48), and decentralized solutions such as AgriBlockIoT have completed performance testing on Ethereum and Hyperledger platforms (39). However, as Behnke and Janssen (5) identified through 16 interviews, successful blockchain application has prerequisites, including process standardization, joint platform construction, and independent governance mechanisms, which partially explains why most studies remain at the pilot stage. A restaurant traceability prototype based on blockchain and food quality data index also demonstrated the feasibility of algorithmically assessing whether food is suitable for consumption (49).

Regarding detection and monitoring capabilities, AI and ML technologies demonstrated higher accuracy than traditional methods, although evidence primarily came from technology validation studies rather than real-world deployments. Pathogen detection systems based on optical sensing and ML achieved detection accuracy of 95% for *Escherichia coli* and *Salmonella enterica* in fresh produce (50). Fruit freshness classification was the most concentrated application scenario, with CNN models based on AlexNet achieving accuracy rates of 98.2%, 99.8%, and 99.3% on three public datasets respectively (35). In the aquatic products sector, a machine vision-based freshness assessment system for common carp achieved classification accuracy of 93.01% through Artificial Neural Networks (ANN) (36). Multi-sensor fusion approaches showed better robustness: Keong et al. (51) proposed a stacked ensemble deep transfer learning model integrating image, odor, and capacitive sensing data, achieving accuracy rates of 91.6 and 93.3% in food ingredient classification and quality determination, respectively. Kaya et al. (52) developed a predictive model with sensor failure tolerance capability for electronic nose applications, improving system reliability in real-world environments. In cold chain logistics scenarios, Huang et al. (37) analyzed blueberry critical control points based on Hazard Analysis and Critical Control Points (HACCP) methodology and constructed ML models using multi-source gas sensor data, with SVM achieving prediction accuracy of 94.01%, outperforming the 85.10% of traditional Arrhenius equation methods. These high accuracy figures require cautious interpretation, as most studies were conducted on standard datasets or in controlled laboratory environments where lighting, background, and sample quality were carefully controlled. In actual supply chain deployments, performance is likely to decline due to environmental variability, sensor degradation over time, inconsistent lighting conditions, and the heterogeneity of real-world food products that differ substantially from curated training samples. The work by Kaya et al. (52) on sensor failure tolerance represents a rare acknowledgment of this gap, but systematic evidence on real-world accuracy degradation remains largely absent from the literature.

IoT technology plays a foundational role in continuous monitoring of food safety, with effects being particularly pronounced when combined with blockchain or AI. Tsang et al. (33) proposed a cold chain risk monitoring system that collects environmental data in real-time through WSN, combining fuzzy logic methods to assess product quality degradation and occupational safety risks for personnel, achieving early warning of potential risks in practical applications at cold chain service companies. The same team's subsequent research integrated IoT with blockchain (53), developing a full-chain traceability shelf-life management system for perishable foods that can automatically record critical control point data and rapidly locate problem sources when anomalies occur. Similar integration patterns have been validated in other studies: an IoT-blockchain framework based on the Hyperledger platform effectively improved data transparency (40), while smart IoT food quality monitoring systems provided low-cost real-time monitoring solutions for home users (54). A food safety early warning system applied in dairy enterprises demonstrated the ability to identify safety risks in advance through association rule mining technology to analyze monitoring data (55).

Consumer and stakeholder acceptance studies provided demand-side supplementary perspectives on technology effects, although such studies were limited in number (*n* = 5). The largest was a consumer survey conducted by Wang and Scrimgeour (56) in New Zealand (*n* = 1,401), which found that perceived incentives and perceived complexity were key factors influencing adoption of blockchain food traceability systems, with consumers showing higher willingness to use blockchain traceability for fresh and imported foods. Tharatipyakul et al. (57), through 10 in-depth interviews and a 350-person questionnaire survey studying the Thai coffee supply chain, found that timeline-based presentation of traceability information was most favored by users, although information accuracy and trade secret protection were major concerns. Panghal et al. (58), through exploratory factor analysis targeting Indian consumers, identified five key factors influencing acceptance: reliability, sustainability, health impact, trust, and switching intentions. These studies consistently indicated that the ultimate effectiveness of technology depends on user adoption willingness rather than solely on technical performance.

### 3.4 Impacts on food loss and waste

Eighteen of the included studies addressed the impacts of digital technologies on food loss and waste (FLW). Compared to the food safety domain, evidence in this area exhibited two characteristics: positive effects predominated in reports but quantitative data was relatively scarce; and technology pathways were mostly indirect, with IoT reducing spoilage through improved environmental monitoring and blockchain optimizing decisions through enhanced information transparency.

IoT monitoring systems applied in cold chain management represent the most direct technical pathway for loss reduction, with related research providing a complete evidence chain from environmental monitoring to loss prevention. Badia-Melis et al. (31) simultaneously deployed RFID tags and WSN in three commercial cold storage facilities (totaling 1,848 cubic meters), achieving three-dimensional spatial temperature mapping of storage environments and estimating energy consumption and product moisture loss based on American Society of Agricultural and Biological Engineers psychrometric data models. This early study established the basic principle that multi-point sensor deployment can identify temperature distribution non-uniformity within storage facilities. The case of UK frozen food company Yumchop Foods provided commercial application validation (32): through real-time temperature and humidity monitoring with IoT sensors, the company successfully identified optimal and non-optimal storage conditions and accordingly established alert and corrective action mechanisms. Recent research has extended monitoring scope to the entire supply chain ecosystem, with Protopappas et al. (34) proposing an IoT service framework covering the full process from packaging to last-mile delivery, providing cost-effective temperature and humidity traceability solutions for perishable agricultural products.

Blockchain technology's contribution to loss reduction is primarily indirect, achieved through improved information sharing and decision coordination. Ahmadkhan et al. (59) used a discrete event simulation model to analyze the impact of blockchain information sharing on a five-tier supply chain network, with results indicating that blockchain can optimize decision-making processes and information infrastructure. This indirect pathway has been demonstrated in multiple cases: research on the Vietnamese coffee supply chain combined green blockchain with the Algorand algorithm, achieving full-chain traceability while reducing system energy consumption (60); a case study of Hungarian sweet potato production demonstrated the combination of near-infrared spectroscopy rapid detection with blockchain traceability (61); and research on strawberry supply chains improved transportation efficiency through the integration of blockchain with IoT containers (62). An Ethereum-based agri-food supply chain framework provided a technical foundation for reducing losses caused by information asymmetry by breaking down information silos and implementing InterPlanetary File System distributed storage (63).

Reports of quantified effects were relatively scarce, but available data provided preliminary references for technology benefit assessment. The SmartNoshWaste framework proposed by Dey et al. (41) integrated blockchain, ML, and cloud computing technologies, with evaluation based on real food data collected from the nosh application showing that the framework achieved a 9.46% reduction in food waste, representing one of the few direct quantified effect reports among included studies. Nagarajan et al. (64) proposed an IoT dynamic routing algorithm for smart city scenarios that reduces product spoilage caused by delays through optimized delivery routes, with evaluation metrics covering tracking accuracy, latency, and execution time. From an overall supply chain perspective, Caldeira et al. (2) used material flow analysis methods to quantify waste distribution across various stages of the EU FSC, finding that cereals, fruits, and vegetables were the most wasted categories and that the consumption stage was the most wasteful for most food categories, pointing to priority areas for digital technology intervention. Research by Verghese et al. (65) in Australia analyzed loss reduction pathways from a packaging perspective, presenting a noteworthy finding: in some cases, reducing food waste requires more rather than less packaging, because improved packaging design can reduce losses through enhanced product protection, improved ventilation, and temperature control. An empirical study of Polish dairy product transportation provided specific data on loss recovery: approximately 0.5% of products (25.08 tons) were lost during transportation over a 2-year period, but a considerable portion had recovery potential due to intact packaging and being safe for consumption (6).

From the perspective of systemic drivers, expert and stakeholder studies identified key conditions for technology effectiveness. Yadav et al. (66) used the Gray Additive Ratio Assessment method to rank performance indicators for IoT traceability systems, finding that sustainable practices were the most important driver, while product tracking and stakeholder behavior showed high sensitivity to system effectiveness. Kumar et al. (67), through feedback from 17 Indian FSC experts and using Interpretive Structural Modeling and network analysis methods, determined that IoT and blockchain are the core enabling technologies driving digital transformation of green FSCs, although inventory management was the least implemented area. Research by Elbasi et al. (38) approached from the agricultural production source, evaluating the performance of 15 ML algorithms in crop prediction, with Bayesian networks achieving classification accuracy of 99.59%, providing technical support for reducing production-end waste through precision planting decisions.

Table 5 presents the distribution of evidence by food category and region. Fruits and vegetables were the most extensively studied, particularly for AI-based freshness detection and IoT cold chain monitoring, reflecting both the high perishability and economic value of these products. Dairy products were well-represented in blockchain traceability research, with multiple cases from European supply chains demonstrating end-to-end tracking capabilities. Meat and poultry studies, including pork in Vietnam and broilers in Thailand, provided important evidence for blockchain cost-effectiveness. In contrast, aquatic products and grains received comparatively limited attention despite their nutritional significance; only one study specifically addressed fish freshness detection, and grain-focused research remained sparse relative to the dietary importance of staples for food-insecure populations. Geographically, evidence concentrated in Asia and Europe, with studies from high-income European countries typically featuring established cold chain infrastructure and reliable connectivity. Evidence from sub-Saharan Africa, where postharvest losses and nutritional insecurity are most severe, was represented by only a single study. This uneven distribution raises questions about the transferability of reported positive effects to low-resource settings where digital technologies may be most needed but face greater implementation barriers.

**Table 5:** Distribution of evidence by food category and geographic region.

Food category	Evidence coverage	Primary technologies	Dominant regions
Fruits and vegetables	Well-documented	IoT cold chain, AI freshness detection	Asia, Europe
Dairy products	Moderate	Blockchain traceability	Europe
Meat and poultry	Moderate	Blockchain traceability	Asia, Europe
Aquatic products	Limited	AI/machine vision	Asia
Grains and staples	Limited	Blockchain, predictive modeling	Asia, Africa
Multiple/cross-category	Extensive	Framework development, integrated systems	Various

### 3.5 Assessment of nutrition-related evidence

The preceding analysis demonstrated that digital technologies have accumulated considerable evidence in food safety assurance and loss reduction. However, when questioning whether these benefits can transmit to the ultimate level of nutritional security, the evidence landscape reveals a significant gap. Among the 46 included studies, only three directly measured nutrition-related outcomes, accounting for 6.5%, a proportion that stands in stark contrast to food safety (58.7%) and loss reduction (39.1%), confirming the research gap proposed in the introduction of this review.

The only empirical study directly measuring nutrient loss came from Bechoff et al. (7), which developed methods and tools for estimating nutritional postharvest losses along food value chains for maize, cowpea, and sweet potato in sub-Saharan Africa. The study combined quantitative and qualitative losses, converting them into nutrient loss quantities and the number of people unable to meet nutritional requirements due to postharvest losses. Their modeling approach revealed that postharvest losses of these three crops resulted in substantial numbers of children under five and pregnant women failing to meet their requirements for vitamin A, iron, and zinc. The research identified multiple factors affecting nutritional postharvest losses, including nutrient density of harvested food materials, handling methods at various value chain stages, extent of pest damage, and differential susceptibility of various nutrients to storage degradation. Vitamin A in orange-fleshed sweet potato, for instance, showed particular vulnerability to light exposure and elevated temperatures during storage, while iron losses in cowpea were primarily associated with insect infestation. These findings underscore the complexity of nutrient retention throughout supply chains and the inadequacy of measuring only physical food quantities. However, it should be noted that the core contribution of this study lies in the development of loss assessment methodology rather than the verification of digital technology intervention effects, leaving the question of whether digital monitoring could mitigate these nutrient losses entirely unanswered. Defraeye et al. (42) attempted to bridge this gap by developing a physics-based digital twin model that integrates real-time sensor data with mechanistic simulations to predict vitamin C and beta-carotene degradation in mango cold chains, yet this work similarly focused on predictive modeling rather than empirical measurement of intervention effects.

The relationship between the remaining 43 studies and nutritional security was entirely indirect and inferential. Although theoretical pathways connecting technology application to nutritional improvement can be hypothesized, none of the included studies empirically tested these connections. No studies measured changes in nutrient retention rates before and after digital technology application, no studies tracked the impact of technology interventions on dietary diversity or nutrient intake, and no studies evaluated the actual effects of supply chain efficiency improvements on the nutritional status of vulnerable populations.

This evidence vacuum is particularly striking given the explicit nutrition-related motivations articulated in many of the included studies. Several papers referenced global hunger statistics and micronutrient deficiency prevalence in their introductions, framing digital technology adoption as a response to these challenges. Yet when it came to outcome measurement, the same studies defaulted to technical performance indicators, supply chain efficiency metrics, or food safety parameters, never closing the loop back to the nutritional justifications that motivated the research. This pattern suggests a systematic disconnect between the stated rationale for digital technology research in food systems and the actual evidence being generated. The factors underlying this disconnect, including disciplinary, methodological, and institutional barriers, will be examined in the Discussion.

The supplementary search conducted in nutrition-focused databases reinforced this finding. Searches in the Cochrane Library and Global Health databases using nutrition-specific terminology combined with digital technology and supply chain terms yielded no additional studies meeting inclusion criteria. Table 6 summarizes the evidence distribution of included studies across three outcome categories. The data clearly shows that existing research is highly concentrated at the food safety and technical efficiency levels, while systematic evidence gaps exist at the nutritional level. This distribution pattern indicates that the research pathway from digital technology application to nutritional security improvement has not yet been established, representing not merely a gap but a fundamental disconnect that urgently needs to be addressed.

**Table 6:** Distribution of outcome types and effect directions in included studies.

Outcome type	Number of studies	Percentage	Positive effects	No significant difference	Not clearly reported
Food safety and quality	27	58.7	24	1	2
Food loss and waste	18	39.1	15	0	3
Nutrition-related outcomes	3	6.5	1	0	2

## 4 Discussion

### 4.1 Core findings: the unverified chain from technology to nutrition

Through systematic review of 46 empirical studies, this scoping review revealed a thought-provoking phenomenon: research on digital technology applications in FSCs has experienced explosive growth, with blockchain, IoT, and AI accumulating considerable positive evidence in traceability, monitoring, and detection domains, yet when we ask whether these technologies can ultimately help people eat more nutritiously, the evidence landscape is almost entirely blank. Among the 27 studies related to food safety, 89% reported positive impacts; among the 18 studies related to loss reduction, 83% showed positive trends; however, only three studies directly measured nutritional outcomes, accounting for just 6.5%. This asymmetric distribution of evidence constitutes the key to understanding the current state of research and points to the most critical gap that needs to be filled in this field.

Compared with recent technology-oriented reviews, this finding offers a different perspective. The reviews by Ellahi et al. (10) and Hassoun et al. (11) comprehensively examined the technical frameworks and application scenarios of blockchain and IoT, while Vasileiou et al. (20) focused on blockchain platform characteristics and the classification of auxiliary technologies. These works made significant contributions at the technical level, but their analytical endpoints typically stopped at supply chain efficiency or food safety, rarely questioning whether technology investments can ultimately translate into improvements in population nutritional status. By placing nutritional security at the core of the analytical framework, this review identified this overlooked gap. Hwalla et al. (68) previously noted that nutritional security should be embedded within all four dimensions of food security, including availability, accessibility, utilization, and stability, yet existing digital technology research primarily focuses on the first two dimensions, with notably insufficient consideration of nutritional utilization and dietary quality. This perhaps explains why the nutritional perspective has long been absent from this field.

The nature of this evidence gap warrants careful characterization. It is not simply that researchers have failed to measure certain outcomes; rather, there appears to be a systematic decoupling between the motivations articulated for digital technology research and the evidence actually generated. Many included studies invoked global hunger statistics and micronutrient deficiency data to justify their work, yet none returned to these nutritional endpoints when evaluating outcomes. This pattern suggests that nutrition serves primarily as a rhetorical frame rather than a genuine research objective in the current literature, a situation that carries implications for both research priority-setting and policy expectations.

### 4.2 Understanding the evidence disconnect

The stark absence of nutrition-related evidence in digital technology research is not coincidental but reflects systematic barriers operating across multiple dimensions. Understanding these barriers is essential for charting a path toward more nutrition-sensitive research in this field.

Disciplinary fragmentation represents a primary obstacle. Research on digital technologies in FSCs is predominantly conducted by scholars in computer science, engineering, and supply chain management, disciplines whose training and publication incentives orient toward technical performance metrics rather than health outcomes. A review of authorship affiliations in included studies reveals that involvement of researchers from nutrition, public health, or food science backgrounds was rare. This disciplinary composition shapes everything from research question formulation to outcome selection. The conceptual frameworks employed in most studies, centered on supply chain efficiency, traceability accuracy, and detection precision, are native to engineering disciplines but largely foreign to nutritional epidemiology. Conversely, nutrition researchers who might bring relevant expertise to technology evaluation often lack familiarity with digital technology capabilities and supply chain dynamics, creating a mutual barrier to collaboration.

Methodological challenges further compound the problem. The temporal mismatch between technology validation and nutrition research is particularly consequential. Digital technology effects on supply chain operations can be observed rapidly, often within days or weeks, making them amenable to the short-term pilot studies that dominate this literature. Nutritional impacts, by contrast, require extended observation periods to manifest at population level, and demand research designs capable of isolating technology effects from the many other factors influencing dietary intake and nutritional status. The quasi-experimental and longitudinal designs necessary for credible nutrition impact evaluation are resource-intensive and fall outside the typical scope of technology development projects. Additionally, current IoT monitoring systems track environmental parameters such as temperature and humidity rather than nutrient content itself. While correlations between storage conditions and nutrient degradation are well-established in food science literature, these relationships have rarely been operationalized in digital monitoring frameworks, creating a measurement gap between what technologies capture and what matters for nutrition.

Institutional incentives also contribute to this disconnect. Technology validation studies with clear quantitative metrics attract publication interest and citation impact more readily than complex intervention studies with ambiguous nutritional endpoints. Research funding in the digital agriculture space has predominantly supported technical innovation rather than downstream impact evaluation. The pressure to demonstrate rapid returns on technology investments creates incentives to measure outcomes that can be assessed quickly and unambiguously, disadvantaging the longer-term, more complex assessments that nutritional impact evaluation requires. These incentive structures are not immutable, but shifting them will require deliberate effort from funders, journals, and research institutions.

### 4.3 Toward nutrition-sensitive technology evaluation

Bridging the identified disconnect requires coordinated action from multiple stakeholders. For policymakers, existing evidence supports prioritizing investment in cold chain IoT and blockchain traceability systems, but policy design must confront the digital divide problem. Research by Mehrabi et al. (23) indicated that the proportion of small-scale farms with access to mobile network services is far lower than that of large-scale farms, and regions with severe yield gaps and food insecurity are precisely those with the poorest network coverage. The barriers extend beyond infrastructure access. Initial capital investment for IoT sensor networks, blockchain platform integration, and data management systems can be prohibitive for small and medium-sized enterprises that constitute the majority of food supply chain actors in developing countries. Ongoing operational costs compound this challenge: sensors require regular calibration and replacement, software platforms demand subscription fees, and data systems need continuous maintenance. Perhaps most critically, smaller operators typically lack the technical expertise to troubleshoot system failures, interpret complex data outputs, or train staff in new procedures. This creates dependence on external support that may be unavailable or unaffordable in resource-limited settings. Without targeted subsidies, capacity-building programs, and simplified technology solutions designed for low-resource contexts, the benefits of digital transformation risk accruing primarily to larger, better-resourced actors. Smallholders and small enterprises, who handle a substantial share of food in developing country supply chains, may be excluded from these benefits. The analysis by De Schutter et al. (69) on the EU Common Food Policy emphasized the need for better coordination between food, agriculture, health, and environmental policies, a governance perspective equally applicable to ensuring that technology investments translate into nutritional improvements.

For researchers, the most urgent task is to incorporate nutrition indicators into study design. This means expanding outcome measurement beyond technical performance to include nutrition-sensitive indicators at multiple levels. At the supply chain level, relevant indicators include nutrient retention rates for specific vitamins and minerals, bioactive compound preservation, and microbiological quality affecting nutrient bioavailability. At the market and household level, dietary diversity scores, food prices and affordability indices, and availability of nutrient-dense foods in retail outlets become important. At the population level, studies should ultimately connect to micronutrient deficiency prevalence and anthropometric measures in vulnerable groups. The selection of indicators should be guided by the specific technology-food-context combination; cold chain IoT interventions for leafy vegetables should prioritize vitamin C and folate retention, while blockchain traceability for animal products might focus on food safety and protein quality.

The digital twin concept proposed by Onwude et al. (70) offers a promising methodological direction, enabling monitoring and prediction of quality evolution throughout the postharvest lifecycle through virtual representation models. Such approaches could potentially be extended to track nutritional changes by integrating environmental monitoring with nutrient degradation kinetics. Such approaches could help bridge the gap between the environmental parameters that current technologies measure and the nutritional outcomes that ultimately matter. Schudel et al. (71), based on collaboration with cold chain stakeholders, compiled a practical roadmap of over 30 postharvest loss reduction measures covering storage temperature optimization, packaging ventilation improvement, and gas composition control, providing actionable frameworks that could be extended to incorporate nutrition-sensitive metrics.

For enterprises, initiating digital transformation from the storage and transportation stage represents a pragmatic choice, as this is where both technology maturity and loss reduction potential are highest. However, industry actors should recognize that demonstrating nutritional benefits, not merely efficiency gains, may increasingly become a requirement for social license and policy support. Partnerships between technology developers and nutrition researchers should be actively cultivated, as such collaborations can generate evidence that serves both commercial and public health objectives.

Research resources should be strategically directed toward contexts where both digital technology potential and nutritional needs are greatest. Geographically, this means prioritizing sub-Saharan Africa and South Asia, where postharvest losses are highest and micronutrient deficiencies most prevalent, yet where current evidence is virtually absent. In terms of food systems, nutrient-dense perishables including fruits, vegetables, and animal-source foods warrant particular attention, as these are both vulnerable to supply chain losses and critical for addressing hidden hunger. Regarding target populations, children under five, pregnant and lactating women, and other nutritionally vulnerable groups who stand to benefit most from improved food access and quality should be the ultimate beneficiaries against whom technology impacts are assessed.

From the perspective of Sustainable Development Goals, the findings of this review are directly relevant to SDG 2 (Zero Hunger) and SDG 12 (Responsible Consumption and Production). Digital technologies, through reducing food loss, have the potential to advance both goals simultaneously. However, as the cross-national comparative study by Ambikapathi et al. (9) demonstrated, although global food system transformation has improved dietary affordability, food systems are still falling short of delivering optimal outcomes in terms of nutrition and health as well as inclusion and equity. Technological progress does not automatically translate into nutritional improvement; conscious policy guidance, institutional arrangements, and deliberate research design are required to realize this potential.

### 4.4 Limitations and future research directions

This review has several limitations that should be acknowledged. The literature search was restricted to English peer-reviewed journal articles, potentially missing enterprise practice reports and local research from developing countries. Considering that digital technology applications are often enterprise-driven and that developing countries are where food loss and nutritional insecurity problems are most severe, future reviews should expand the literature scope to include technical reports from international organizations such as FAO and the World Bank, as well as non-English literature. The policy analysis by Thow et al. (72) on the United Nations Decade of Action on Nutrition indicated that the connections between trade, investment, and nutrition vary significantly across different country contexts, a heterogeneity that tends to be underestimated in reviews dominated by English literature.

At the study design level, included studies primarily consisted of case studies and technology validation, lacking comparative studies with control groups, which creates uncertainty in effect attribution. The high proportion of studies reporting positive effects, 89% for food safety and 83% for loss reduction, may partially reflect publication bias, as studies finding null or negative results are less likely to reach publication. This positivity bias is common in emerging technology fields and suggests that the actual effectiveness of digital technologies may be somewhat less consistent than the included literature implies. Future primary research should more frequently employ quasi-experimental designs establishing intervention and control groups in real supply chain environments, and journals should be encouraged to publish null findings to provide a more balanced evidence base. A feasible approach would pair comparable supply chains, implementing technology interventions in some while others continue conventional practices. Outcome measurement should span multiple levels, from supply chain performance and product nutrient retention at delivery to downstream availability and pricing where possible. Mixed-methods components including operator interviews would help explain implementation success or failure. Such designs demand collaboration between technology researchers, nutrition scientists, and supply chain operators, partnerships that remain rare but essential for bridging the technology-to-nutrition evidence gap.

The scarcity of nutrition indicators in existing research represents the core gap identified by this review, and addressing this gap should be a priority for future research design. Al Shoffe and Johnson (73) noted that developing reliable postharvest loss prediction models is crucial for supporting decision-making, and validation of such models requires long-term tracking data rather than relying solely on cross-sectional cases. A feasible starting point would be adding proxy indicators related to nutritional degradation to existing cold chain monitoring systems, such as specific gas concentrations or cumulative light exposure, parameters that have known associations with losses of certain vitamins. The review by Revelou et al. (74) on machine learning applications in food safety monitoring suggested that the combination of non-destructive detection technologies with AI provides technical possibilities for real-time nutritional assessment, an avenue that deserves exploration.

Geographic coverage also warrants attention. Included studies primarily came from middle- and high-income countries in Asia and Europe, with notably insufficient research from nutritionally vulnerable regions such as sub-Saharan Africa. Considering that infrastructure conditions and supply chain characteristics in these regions differ significantly from those in developed countries, the external validity of existing evidence is questionable. Future research should prioritize application scenarios in low-income countries and evaluate the actual possibilities for smallholder farmers to benefit from digital technologies. Von Braun et al. (75), in discussing action challenges for reducing food loss, emphasized that technical solutions must be adapted to local conditions to be effective, a principle particularly important for bridging the digital divide.

## 5 Conclusions

This scoping review systematically examined the evidence on digital technology applications in FSCs, attempting to answer a seemingly simple yet long-overlooked question: can these technologies actually help people eat more safely and more nutritiously? The answer is complex. Blockchain, IoT, and AI have demonstrated clear value in improving traceability efficiency, enhancing environmental monitoring, and increasing detection accuracy, with positive evidence in food safety and loss reduction continuing to accumulate. However, when we shift our focus from technical performance to ultimate nutritional outcomes, the evidence landscape is almost entirely blank. This finding is not intended to negate the potential of digital technologies, but rather to remind us that no automatic transmission mechanism exists between technology investment and nutritional improvement. Bridging this gap requires the joint efforts of researchers, policymakers, and enterprises. For a research field that envisions “farm to fork” and aims for sustainable development, if it perpetually remains at the level of supply chain efficiency without questioning what changes actually occur on people's tables, then this vision remains incomplete. Future research and practice need to explicitly incorporate nutritional security into evaluation frameworks. Only then can digital technologies truly become a bridge connecting sustainable food systems with human health, rather than merely serving as tools for improving commercial efficiency.
